# Mutations in the D1 domain of von Willebrand factor impair their propeptide-dependent multimerization, intracellular trafficking and secretion

**DOI:** 10.1186/s13045-015-0166-9

**Published:** 2015-06-20

**Authors:** Jie Yin, Zhenni Ma, Jian Su, Jiong-Wei Wang, Xiaojuan Zhao, Jing Ling, Xia Bai, Wanyan Ouyang, Zhaoyue Wang, Ziqiang Yu, Changgeng Ruan

**Affiliations:** Collaborative Innovation Center of Hematology, MOH Key Lab of Thrombosis and Hemostasis, Jiangsu Institute of Hematology, the First Affiliated Hospital, Soochow University, Suzhou, 215006 China; Department of Surgery, National University of Singapore; Cardiovascular Research Institute (CVRI), National University Heart Centre Singapore (NUHCS), National University Health System, Singapore, 999002 Singapore; Department of Hematology and Oncology, Children’s Hospital of Soochow University, Suzhou, 215003 China

**Keywords:** von Willebrand disease, VWF propeptide, VWF gene mutation, VWF multimerization, ER retention

## Abstract

We identified three novel mutations (p.Gly39Arg, p.Lys157Glu, p.Cys379Gly) and one previously known mutation (p.Asp141Asn) in the von Willebrand factor propeptide from three von Willebrand disease patients. All four mutations impaired multimerization of von Willebrand factor, due to reduced oxidoreductase activity of isomeric propeptide. These mutations resulted in the endothelial reticulum retention and impaired basal and stimulated secretions of von Willebrand factor. Our results support that the mutations in the D1 domain lead to defective multimerization, intracellular trafficking, and secretion of von Willebrand factor and result in bleeding of patients.

**Electronic supplementary material:**

The online version of this article (doi:10.1186/s13045-015-0166-9) contains supplementary material, which is available to authorized users.

## Findings

Von Willebrand factor propeptide (VWFpp), composed of D1 and D2 domains, is necessary for the multimerization, intracellular trafficking, and secretion of the factor [1–3]. Few mutations in the D1 domain of VWFpp have been reported, and the pathogenic nature of these mutations remains largely unknown [4, 5].

We found three novel mutations (p.Gly39Arg, p.Lys157Glu, p.Cys379Gly) and one previously known mutation (p.Asp141Asn) in the VWFpp from three von Willebrand disease (VWD) patients (Additional file 1). The proband 1 (P1) and proband 2 (P2) were type 3 VWD, while the proband 3 (P3) was type 1 VWD [6]. The laboratory results of these patients and their family members are summarized in Table 1. P1 and P2 presented undetectable von Willebrand factor (VWF) multimer, and P3 exhibited light multimer pattern, compared to normal plasma (NP) (Additional file 2: Figure S1). All mutations are located in the D1 domain of VWFpp.

**Table 1:** Phenotypic and genetic features of three unrelated VWD families

Family	Members	VWF mutation	Genotype	APTT ratio	PT ratio	TT ratio	VWF: Ag	VWF: RCo	FVIII: C	BS
				(sec)	(sec)	(sec)	(IU/dL)	(IU/dL)	(IU/dL)	
F1	Proband P1	G39R D141N	Compound heterozygous	2.24	1.07	0.97	1.0	2.3	2.0	6
F1	Father P1F	D141N	Heterozygous	1.09	1.15	1.24	48.4	50.2	73.5	0
F1	Mother P1M	G39R	Heterozygous	1.12	1.05	0.91	30.3	36.4	50.6	1
F2	Proband P2	K157E C1165R	Compound heterozygous	1.95	1.04	0.89	3.0	2.1	3.0	6
F2	Father P2F	K157E	Heterozygous	0.94	1.02	0.98	87.2	80.3	97.8	1
F3	Proband P3	C379G	Heterozygous	1.50	1.17	1.07	8.0	5.9	13.1	9
F3	Brother P3B	C379G	Heterozygous	1.21	1.00	0.90	14.3	13.6	25.3	3
Normal range	–	–	–	0.82–1.18	0.86–1.14	0.80–1.20	50–160	50–120	50–150	0–3 or 0–5^a^

*PT* prothrombin time, *APTT* activated partial thromboplastin time, *TT* thrombin time, *VWF:Ag,* von Willebrand antigen, *VWF:RCo* von Willebrand factor ristocetin cofactor activity, *FVIII:C* factor VIII coagulant activity, *BS* bleeding scores

^a^0–3 in male, 0–5 in female

**Fig. 1:**
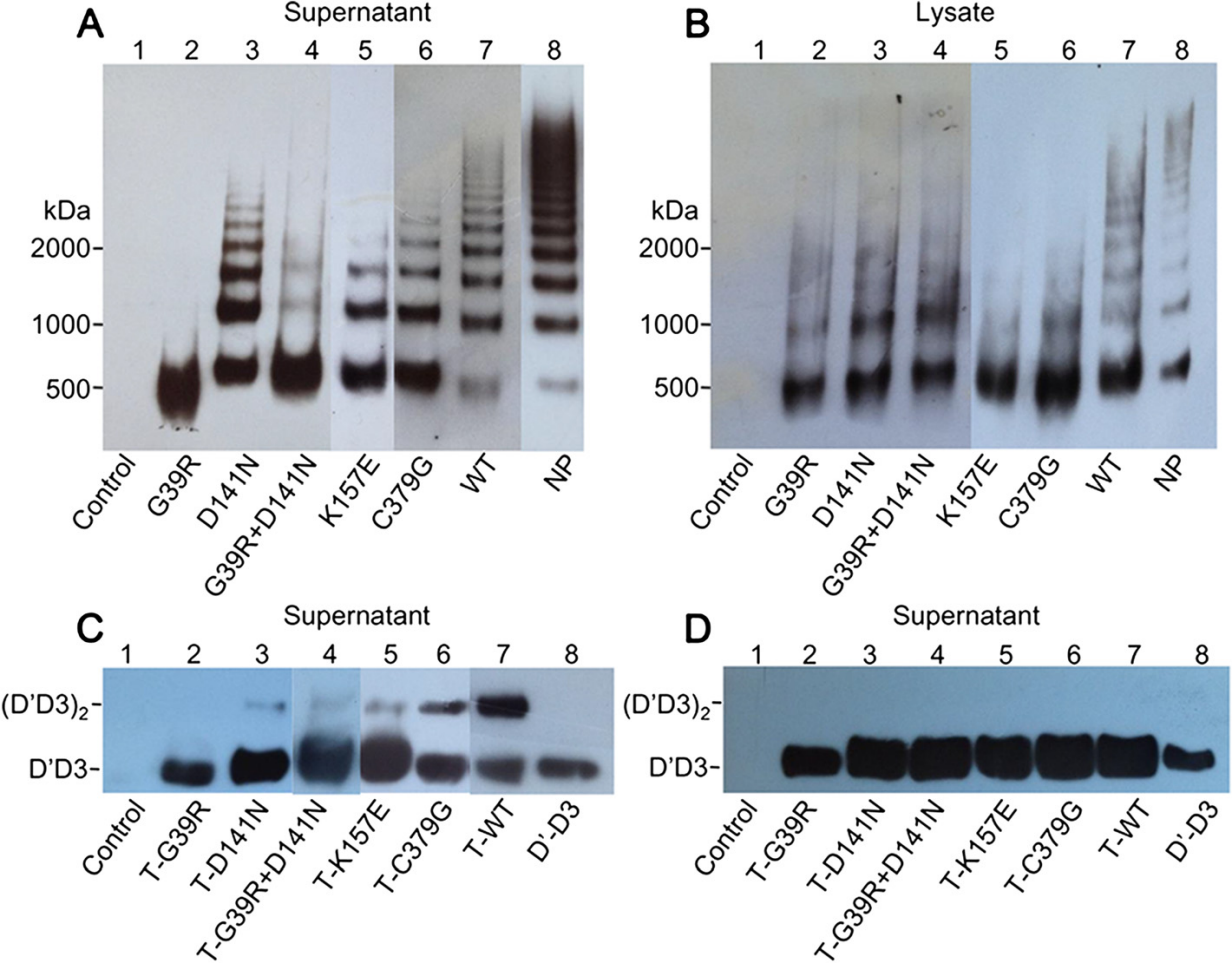
Defective multimerization of mutant VWF and reduced dimerization of mutant truncated VWF. **a** HEK293 cells were transiently transfected by equal WT or mutant full-length VWF plasmid. Seventy hours after transfection, the supernatant of cells were collected for multimer analysis. In order to detect whether the mutations have abnormal multimer pattern, we measured equal VWF antigen of the mutations, wild type, and NP. Lane 1 is the negative control of the mimic empty vector. Lanes 2–6 show the supernatant obtained from VWF mutant transfections and indicate decreases in high to medium-sized multimers to varying degrees. Exclusively, dimers formed with the Gly39Arg mutant (lane 2). The ability to form multimers was partly recovered by co-transfection of Gly39Arg and Asp141Asn (lane 4). **b** In the lysates of transfected cells, a similar decrease in the formation of VWF multimers was seen in the variants. However, WT-VWF exhibited a multimer pattern as observed for normal plasma. **c** HEK293 cells were transiently transfected by equal WT or mutant truncated VWF. In the media of transfected cells, dimerizations of truncated recombinant VWF were detected by Western blotting under the non-reducing condition. Lane 1 shows the empty pSecTag2/Hygro B vector. Dimers were absent in the Gly39Arg mutant (lane 2) while lanes 3–6 tapered in the dimer to different degrees. **d** In the supernatant of the transfected cells, the truncated VWF mutations and WT D1D2D′D3 fragment were examined under the reducing condition, and only D′D3 monomers were found

To determine whether and how these mutations impair VWF expression and function, we transiently transfected the human embryonic kidney 293 cells (HEK293) with wild type (WT) or VWF mutant constructs and analyzed VWF multimer (Fig. 1a,b). In the supernatant, the WT-VWF showed a full range of multimers similar to NP. Asp141Asn, Lys157Glu, and Cys379Gly mutants each exhibited different degrees of the loss of large- and medium-sized multimers. We also co-transfected Asp141Asn and Gly39Arg mutants and found that a partial VWF protein multimerization was readily detectable. When mutant and WT-VWF were co-transfected into HEK293 cells, the abnormal VWF multimers were all restored (Additional file 3: Figure S2). We examined cell lysates for VWF multimer and found that multimerization from all VWF mutations were inhibited in different levels as showed in the media. To determine the underlying mechanisms of defective VWF multimerization, we employed a previously established dimerization model using D1D2D′D3 fragment of VWF [7, 8]. In this model, the decreased dimerization of D1D2D′D3 indicates the reduced oxidoreductase of mutant VWFpp. Under the non-reducing condition (Fig. 1c), truncated WT (T-WT) VWF formed both D′D3 monomers and dimers in the supernatant. The dimerizations of truncated four variants (T-Gly39Arg, T-Asp141Asn, T-Lys157Glu, and T-Cys379Gly) were reduced, compared to that observed with T-WT. Co-transfection of the T-Gly39Arg and T-Asp141Asn resulted in a modest restoration of dimer formation compared to the single transfection of truncated Gly39Arg. This result indicates that the impaired multimerization is caused by decreased oxidoreductase activity of the isomeric propeptide. Under the reducing condition (Fig. 1d), D′D3 dimers were reduced to D′D3 monomers in both mutant and wild type D1D2D′D3 fragment.

The VWF antigen was measured in the conditioned media and cell lysates of the transiently transfected HEK293 cells (Additional file 4: Figure S3). The expressions of mutant Gly39Arg or Asp141Asn in the supernatant were significantly impaired, at the level of 2.8 ± 0.3 % to 2.5 ± 0.1 % of the WT. However, the expressions of mutant Lys157Glu or Cys379Gly product in the supernatant were less severely decreased at the levels of 26.0 ± 4.1 % and 22.4 ± 3.8 % of the WT. In co-transfection of Gly39Arg and Asp141Asn constructs, only 2.6 ± 0.1 % VWF was detectable in the supernatant, implying that both mutants do not mutually rescue each other from their defects. We also co-transfected the WT and each mutant (Gly39Arg, Asp141Asn, Lys157Glu, or Cys379Gly), and 59.3 ± 4.3 %, 57.3 ± 8.9 %, 86.6 ± 7.1 %, or 33.9 ± 1.2 % of WT were detected, respectively. This suggests that four mutants can be partially restored by the WT. In the cell lysates of single or co-transfected variants, the VWF levels did not changed dramatically.

We found that the level of VWF antigen in the supernatant of HEK-293 cells expressing WT-VWF increased by 2.28-fold after phorbol 12-myristate-13-acetate (PMA) stimulation. However, cells expressing four mutations exhibited no significant changes in VWF antigen secretion upon PMA stimulation (Additional file 5: Figure S4).

**Fig. 2:**
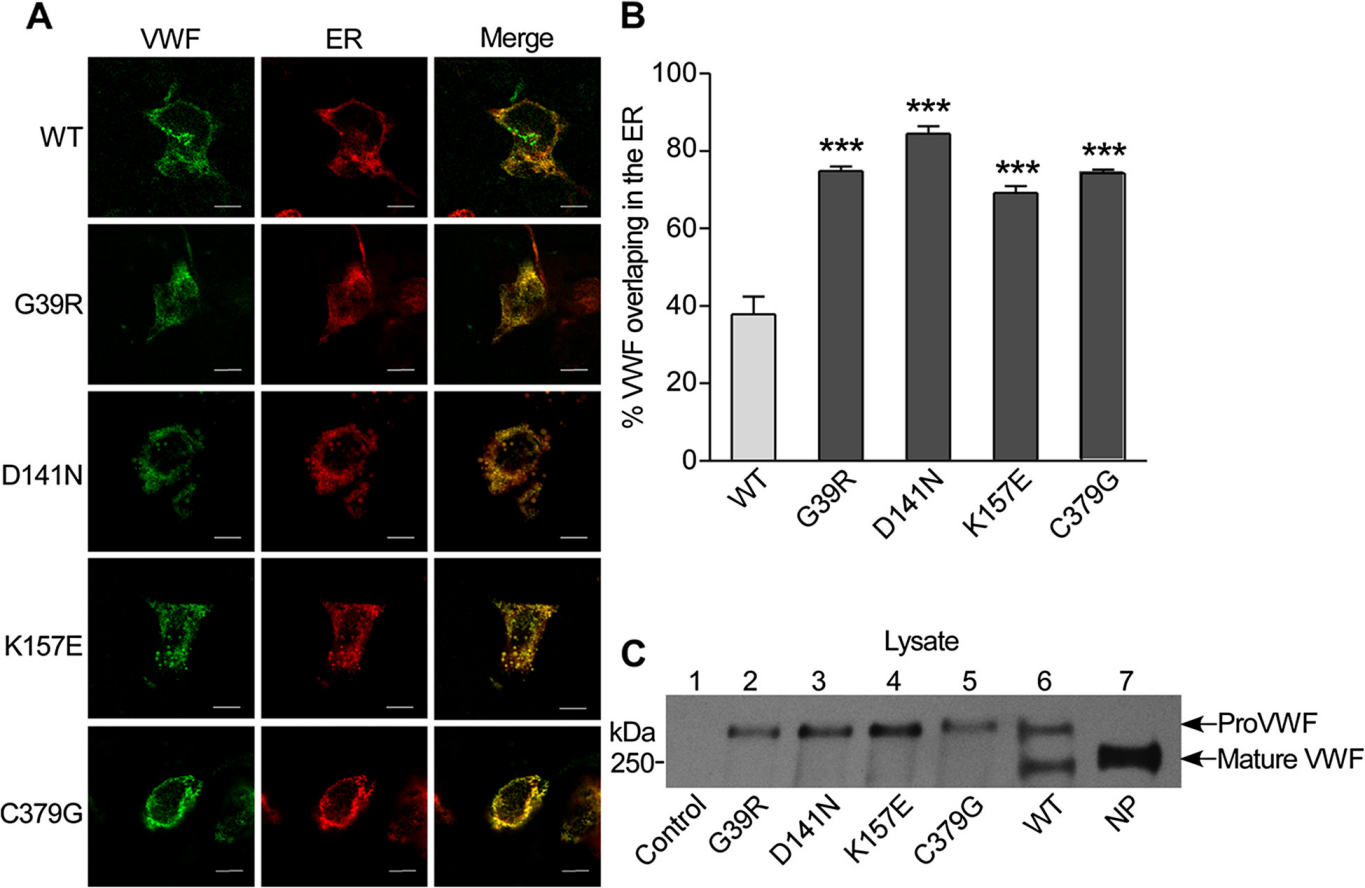
Mutant VWF is retained in the ER. **a** HEK293 cells were transfected with plasmids expressing WT or mutant VWF and then stained for VWF (*green channel*, *left panel*). PDsRed2-ER Vector was also co-transfected into cells to delineate the endoplasmic reticulum (*middle panel*, *red*). The scale bar is 10 μm. The *right panel* shows the merged confocal images of the first two panels. **b** *Bar graph* represents VWF and the ER overlapping as a percentage of total VWF in cells (*n* = 3 independent experiments). All values are mean ± SD. ***p < 0.001 compared to WT (one-way ANOVA). **c** After HEK293 cells were transiently transfected with WT or mutant full-length VWF, cell lysates were reduced and analyzed by Western blotting. The empty vector control is shown in lane 1. The bands of pro-VWF are predominant in the lysates of VWF mutant variants (lanes 2–5). Lane 6 shows both pro-VWF and mature VWF in the lysate of WT, and lane 7 shows only mature VWF in normal plasma

By immunofluorescent staining of VWF proteins (Fig. 2a), we found that VWF formed green granules in cells. Only 37.8 ± 9.3 % WT-VWF was observed in the endoplasmic reticulum (ER), while 74.8 ± 2.5 % Gly39Arg, 84.6 ± 3.8 % Asp141Asn, 69.2 ± 3.6 % Lys157Glu, and 74.3 ± 1.8 % Cys379Gly mutants were detected in the ER (Fig. 2b). In the cell lysates, the bands of pro-VWF were predominant, and mature VWF was hardly detectable in the cells expressing Gly39Arg, Asp141Asn, Lys157Glu, and Cys379Gly, compared to that of WT-VWF (Fig. 2c). These results indicate that the mutant VWF are retained in the ER and fail to be transported to the Golgi, resulting in the decreased VWF secretion.

In summary, four mutations in the D1 domain of VWF impair the multimerization of VWF by directly downregulating the oxidoreductase of the propeptide, disrupt the transport of VWF from the ER to the Golgi, and inhibit the basal and regulated secretion of VWF. These defects contribute to the quantitative loss of VWF, leading to the bleeding diathesis of VWD patients.

## Additional files

Additional file 1:
**Supplementary data.** Detailed methods and materials are shown. Additional file 2: Figure S1. Plasma VWF multimers in three unrelated VWD patients and their family members. (A) Plasma samples were assessed by 1.3 % SDS-agarose gel electrophoresis and Western blotting, and the image was taken after exposure for 3 min. Normal plasma (NP) was shown in lane 4, 7 and 10 as a positive control. VWF multimer structures were absent in lane 1 (P1) and lane 5 (P2), while P3 and P3B presented very light multimer bands. (B) The image was taken after 6-minute exposure. VWF multimer patterns of P3 and P3B were normal while those of P1 and P2 were still absent. (C) Schematic diagram of VWF. Arrows indicate the locations of four mutations in the D1 domain on the whole pro-VWF. Additional file 3: Figure S2. Restored VWF multimerization of co-expression of mutant and WT-VWF. HEK293 cells were transiently transfected by WT and mutant full-length VWF plasmid in ratio of 1:1. VWF multimers present normal pattern in all co-expression of mutant and WT-VWF, compared to that observed with WT-VWF. Additional file 4: Figure S3. Decreased basal secretion of mutant rVWF. HEK293 cells were transiently transfected by equal WT or mutant full-length VWF plasmid in single transfection. In co-transfection, two plasmids were transfected in ratio of 1:1, and added up to the equivalent plasmid of single transfection. VWF levels were determined in conditioned media and cell lysates from HEK293 cells transfected with single expressing vector or co-expressing vectors in duplicate. Each bar represents the average values of three separate transfections. VWF levels were measured by ELISA and are shown as percentages relative to WT-VWF in media or lysates. The left side of the short vertical line represents single-transfections while the right side represents co-transfections. Additional file 5: Figure S4. Decreased regulated secretion of mutant rVWF. Forty-eight hours post-transfection, HEK293 cells were rinsed and changed to release media with or without PMA (control). After further 60 min incubation, VWF levels were determined by ELISA in the release media and cell lysates. Each bar represents VWF secretion in the medium as a percentage of total VWF (medium plus lysate). Error bars indicate standard deviation of triplicate samples. The numbers above the bars indicate the fold increase of stimulated release compared with the control samples.

## Abbreviations

VWD: von Willebrand disease
VWF: von Willebrand factor
ER: endoplasmic reticulum
VWFpp: VWF propeptide
P1: proband 1
P2: proband 2
P3: proband 3
NP: normal plasma
WT: wild type
PMA: Phorbol 12-myristate-13-acetate
