# Supplementary material for: Mutations in the D1 domain of von Willebrand factor impair their propeptide-dependent multimerization, intracellular trafficking and secretion
Source: J Hematol Oncol. 2015 Jun 20;8:73. doi: 10.1186/s13045-015-0166-9 (PMC4487848; doi:10.1186/s13045-015-0166-9)
Supplement: Additional file 1: — Supplementary data. Detailed methods and materials are shown. [file 13045_2015_166_MOESM1_ESM.docx]

**Supplementary** **data**

**Blood samples and laboratory assays**

This study was approved by the Ethics Committees of Jiangsu Institute of Hematology, and informed consent was obtained from all participants. Whole blood was collected with tubes containing 3.8% citrate sodium. Platelet-poor plasma was prepared by centrifugation for 5 min at 1,200g. VWF: Ag and VWF: RCo were analyzed by enzyme-linked immunosorbent assay (ELISA). FVIII coagulant activity was measured by a one-stage method. Prothrombin time (PT), activated partial thromboplastin time (APTT) and thrombin time (TT) were estimated with a STA Compact automated analyzer (Diagnostica Stago, France). As for the VWF multimer analysis, plasma samples were assessed by 1.3% SDS-agarose gel electrophoresis under the non-reducing condition. When the gel was dry in air, it was immunostained with polyclonal rabbit anti-human VWF/HRP (Dako, Denmark), and visualized using the Western Bright ECL. Three patients were diagnosed as VWD according to the 2006 classification of von Willebrand disease [6]. Bleeding scores of all participants were evaluated by Bleeding Assessment Tool (<http://www.isth.org/members/group.aspx?id=100549>).

**Mutation detection**

Genomic DNA was isolated from whole blood using the QIAamp DNA Blood Mini Kit (Qiagen, Hilden, Germany) according to the manufacturer’s instructions. DNA sequences of the whole VWF gene including all 52 exons, intron-exon boundaries, and the 2.8 kb upstream of exon 1 were amplified by PCR. The primers are available upon request. Bidirectional sequence of the VWF gene was carried out using BigDye Terminator Cycle Sequencing Ready Reaction Kit in an ABI Prism 3130 Genetic Analyzer (Applied Biosystems). Sequence data were analyzed and compared to VWF reference sequence [GeneBank: NC_000012.1].

**Plasmid construction**

Plasmid pSVHvWF1 containing full-length human VWF cDNA was kindly provided by Dr. Evan Sadler (Washington University School of Medicine, MO, USA). Mutations (Gly39Arg, Asp141Asn, Lys157Glu, and Cys379Gly) were introduced into wild type VWF plasmid by site-directed mutagenesis using Phusion DNA polymerase (New England Biolabs, MA, USA) and specific oligonucleotide primers (Additional file 1: Table S1). The mutations in the VWF expression plasmids were verified by sequencing.

VWF fragments of D1D2D’D3 were amplified (corresponding to amino acids 23-1241) using WT pSVHvWF1 or pSVHvWF1 containing mutations as templates. VWF fragments were then inserted into the expression vector pSecTag2/Hygro B (Invitrogen, CA, USA). Plasmid pSecTag2B containing D’D3 was constructed with the same method. Plasmid alterations were verified by sequencing.

**Cell culture and transfection**

HEK293 cells (Cell Bank of Chinese Academy of Science, Shanghai, China) were cultured in Dulbecco's Modified Eagle's medium (Thermo Scientific, Beijing, China) supplemented with 10% fetal calf serum, 10mM HEPES, and 2mM L-glutamine (Sigma-Aldrich, MO, USA). HEK293 cells were transiently transfected using TurboFect transfection reagent (Thermo Scientific, Lithuania).

**Expression of truncated VWF**

Truncated WT-VWF (D1D2D’D3 domain), mutant-VWF fragments, and D’D3 domains were produced from transiently transfected HEK293 cells. Conditional media were collected 72 h after transfection and electrophoresed through 8% Tris-glycine gel under the non-reducing and reducing condition, respectively. VWF fragments were immunostained with polyclonal rabbit anti-human VWF/HRP (Dako, Denmark) and were visualized with Western Bright ECL (Advansta, CA, USA).

**Structural analysis of VWF**

Conditional media were collected 72 h after HEK293 cells were transfected with full-length WT or mutant VWF. Cells were washed once with phosphate-buffered saline and then lysed at 4 °C for 16 h in Passive Lysis Buffer (Promega, WI, USA) containing the protease inhibitor mixture Complete TM with EDTA (Roche Diagnostics, Mannheim, Germany). Cell lysates were centrifuged for 5 min at 10,000g. The supernatants were supplemented with phenylmethylsulfonyl fluoride (Sigma-Aldrich, MO, USA) at a final concentration of 100μM. VWF multimers were analyzed in the media and cell lysates under non-reducing condition. Cell lysates were reduced in sample buffer containing 20 mM dithiothreitol and separated on 6% Tris-glycine gel, then immunostained with polyclonal rabbit anti-human VWF/HRP (Dako, Denmark) of a1:1000 dilution, and visualized using the Western Bright ECL.

**Basal and regulated secretion of VWF**

In the basal secretion of VWF, the supernatant of HEK293 cells 24 h after transfection were removed and cells were incubated in fresh culture medium for 48 h. The media and cell lysates were collected to determine the VWF antigen. To analyze the regulated secretion of VWF, 48 h after transfection, HEK293 cells were cultured in the media with PMA (Sigma-Alorich, Saint Louis, MO, USA) or vehicle (dimethylsulfoxide). VWF antigen was measured in the media and cell lysates by ELISA.

**Confocal immunofluorescence microscopy**

HEK293 cells were transfected with VWF plasmid andPDsRed2-ER Vector (Clontech, CA, USA) at a 1:1 ratio. Seventy-two hours after transfection, cells were fixed with 4% paraformaldehyde, and permeabilized by 0.1% triton X-100 (Sangon, Shanghai, China). After incubation with 5% bovine serum albumin, the cells were incubated with polyclonal rabbit anti-human VWF antibody (Dako, Denmark) and then with Alexa Flour 488-conjugated secondary antibody (Invitrogen, CA, USA). Fluorescent images were collected on an Olympus FV1000 MPE-shared confocal microscope. Images were analyzed using FV10-ASW 4.1 Viewer and processed using image-Pro plus 6.0.

**Table S1 Oligonucleotide primers used for site-direct mutagenesis of four mutations**

| Primer designation | Exon No. | Primer Sequence 5’-3’ |
| --- | --- | --- |
| G39R-F | 3 | TTTTCAGAAGTGACTTCGTCAACACCTTTG |
| G39R-R | 3 | CTTCTGAAAAGGCTGCATCGGGCCGT |
| D141N-F | 5 | GGATCAATGGCAGCGGCAACTTTCA |
| D141N-R | 5 | CCATTGATCCTGGCCACAAA GCCA |
| K157E-F | 5 | TCAACGAGACCTGCGGGCTGTGTGGCAA |
| K157E-R | 5 | GTCTCGTTGAAGTATCTGTCTGACAGCAGG |
| C379G-F | 10 | GATCGGCAGCAATGAAGAATGTCC |
| C379G-R | 10 | CTGCCGATCCACTGGCTGTTTC |

**Figure legends**

**Figure S1: Plasma VWF multimers in three unrelated VWD patients and their family members**

(A) Plasma samples were assessed by 1.3% SDS-agarose gel electrophoresis and western blotting, and the image was taken after exposure for 3 min. Normal pooled plasma (NP) was shown in lane 4, 7 and 10 as a positive control. VWF multimer structures were absent in lane 1 (P1) and lane 5 (P2), while P3 and P3B presented very light multimer bands. (B) The image was taken after 6-minute-exposure. VWF multimer patterns of P3 and P3B were normal while those of P1 and P2 were still absent. (C) Schematic diagram of VWF. Arrows indicate the locations of four mutations in the D1 domain on the whole pro-VWF.

**Figure S2: Restored VWF multimerization of co-expression of mutant and WT-VWF**

HEK293 cells were transiently transfected by WT and mutant full-length VWF plasmid in ratio of 1:1. VWF multimers present normal pattern in all co-expression of mutant and WT VWF, compared to that observed with WT-VWF.

**Figure S3: Decreased basal secretion of mutant rVWF**

HEK293 cells were transiently transfected by equal WT or mutant full-length VWF plasmid in single transfection. In co-transfection, two plasmids were transfected in ratio of 1:1, and added up to the equivalent plasmid of single transfection. VWF levels were determined in conditioned media and cell lysates from HEK293 cells transfected with single expressing vector or co-expressing vectors in duplicate. Each bar represents the average values of three separate transfections. VWF levels were measured by ELISA and are shown as percentages relative to WT-VWF in media or lysates. The left side of the short vertical line represents single-transfections while the right side represents co-transfections.

**Figure S4: Decreased regulated secretion of mutant rVWF**

Forty-eight hours post-transfection, HEK293 cells were rinsed and changed to release media with or without PMA (control). After further 60 min incubation, VWF levels were determined by ELISA in the release media and cell lysates. Each bar represents VWF secretion in the medium as a percentage of total VWF (medium plus lysate). Error bars indicate standard deviation of triplicate samples. The numbers above the bars indicate the fold increase of stimulated release compared with the control samples.
