# Supplementary figures and images for: Mutations in the D1 domain of von Willebrand factor impair their propeptide-dependent multimerization, intracellular trafficking and secretion
Source: J Hematol Oncol. 2015 Jun 20;8:73. doi: 10.1186/s13045-015-0166-9 (PMC4487848; doi:10.1186/s13045-015-0166-9)

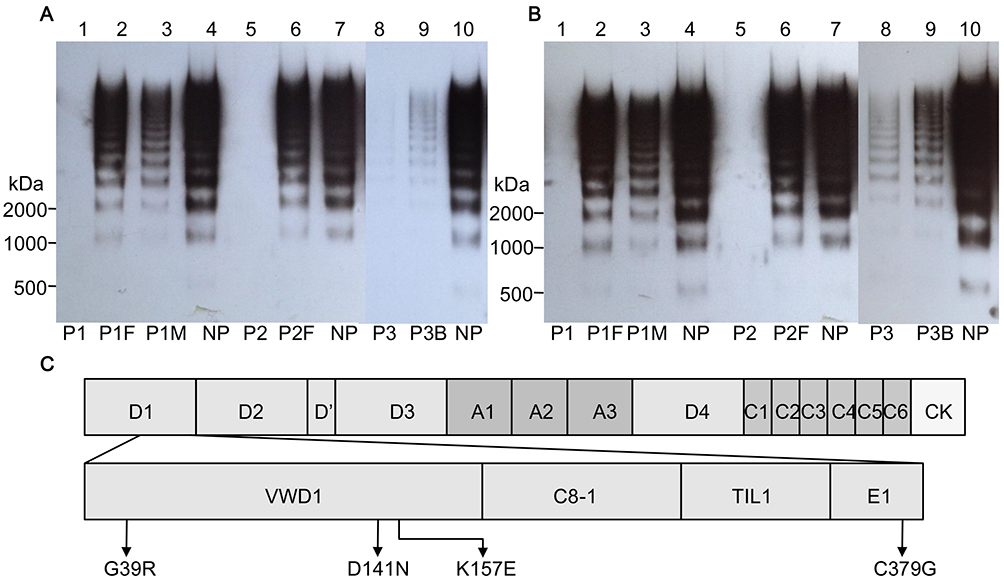

Supplement: Additional file 2: Figure S1. — Plasma VWF multimers in three unrelated VWD patients and their family members. (A) Plasma samples were assessed by 1.3 % SDS-agarose gel electrophoresis and Western blotting, and the image was taken after exposure for 3 min. Normal plasma (NP) was shown in lane 4, 7 and 10 as a positive control. VWF multimer structures were absent in lane 1 (P1) and lane 5 (P2), while P3 and P3B presented very light multimer bands. (B) The image was taken after 6-minute exposure. VWF multimer patterns of P3 and P3B were normal while those of P1 and P2 were still absent. (C) Schematic diagram of VWF. Arrows indicate the locations of four mutations in the D1 domain on the whole pro-VWF. [file 13045_2015_166_MOESM2_ESM.tif]

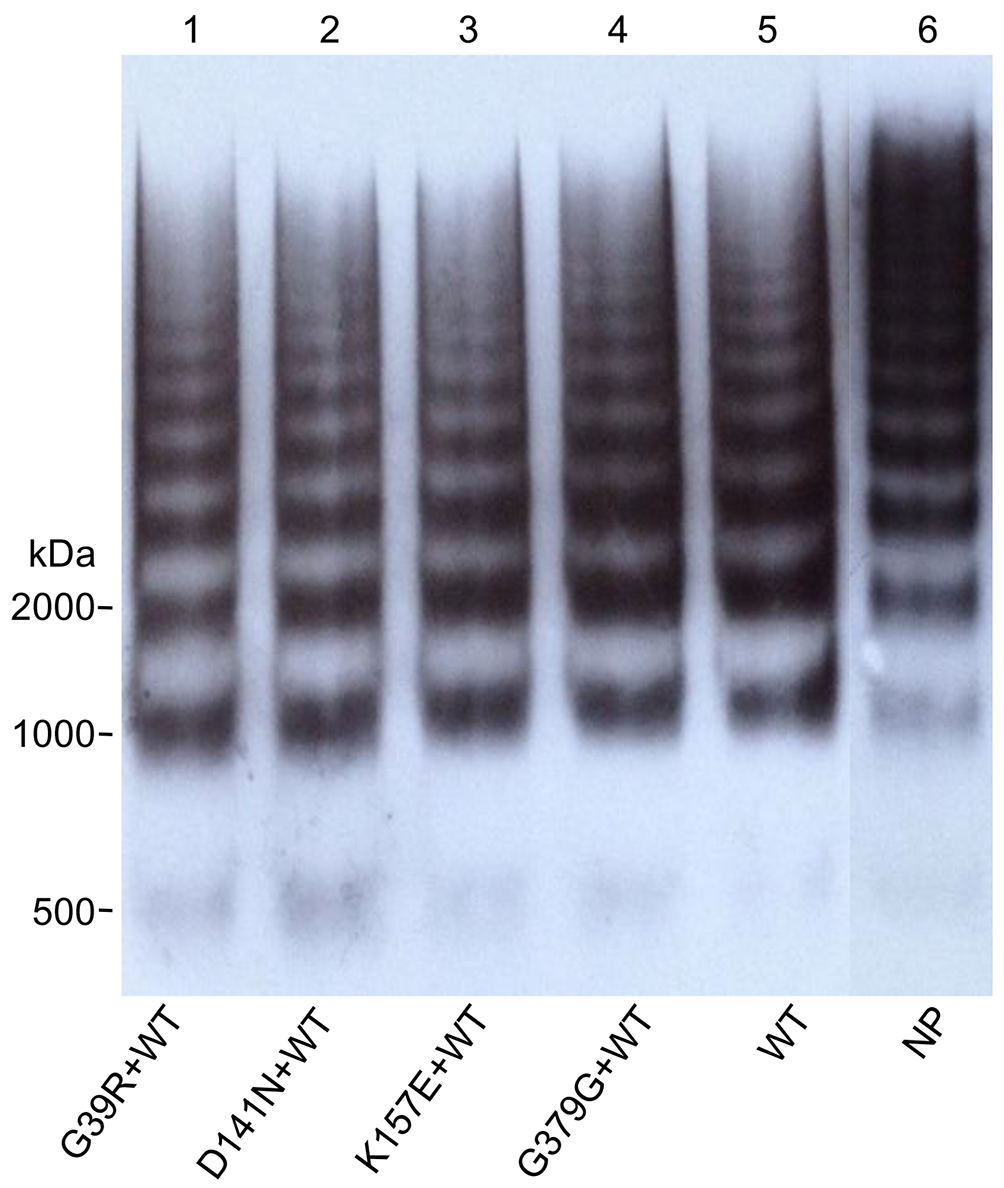

Supplement: Additional file 3: Figure S2. — Restored VWF multimerization of co-expression of mutant and WT-VWF. HEK293 cells were transiently transfected by WT and mutant full-length VWF plasmid in ratio of 1:1. VWF multimers present normal pattern in all co-expression of mutant and WT-VWF, compared to that observed with WT-VWF. [file 13045_2015_166_MOESM3_ESM.tif]

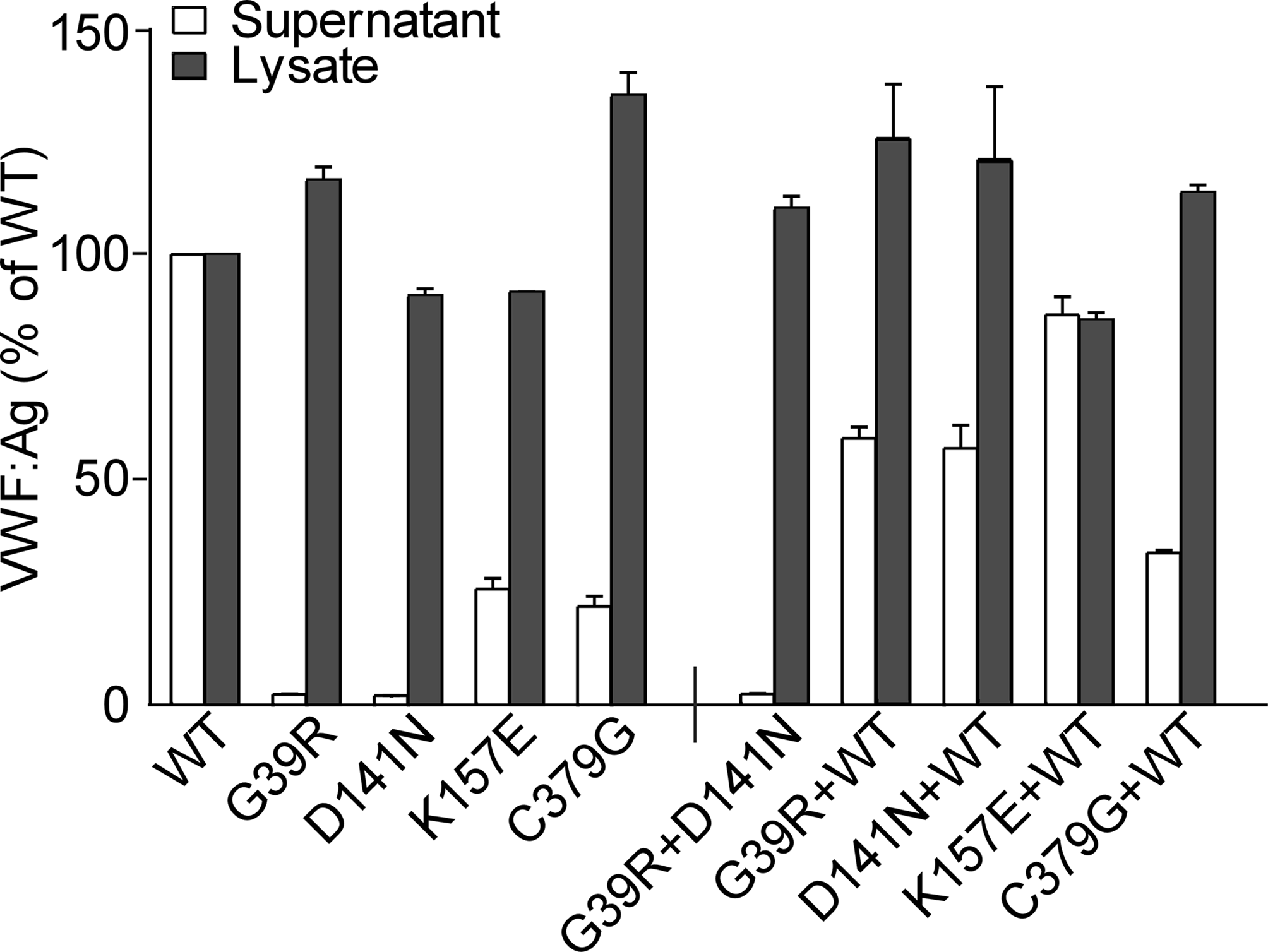

Supplement: Additional file 4: Figure S3. — Decreased basal secretion of mutant rVWF. HEK293 cells were transiently transfected by equal WT or mutant full-length VWF plasmid in single transfection. In co-transfection, two plasmids were transfected in ratio of 1:1, and added up to the equivalent plasmid of single transfection. VWF levels were determined in conditioned media and cell lysates from HEK293 cells transfected with single expressing vector or co-expressing vectors in duplicate. Each bar represents the average values of three separate transfections. VWF levels were measured by ELISA and are shown as percentages relative to WT-VWF in media or lysates. The left side of the short vertical line represents single-transfections while the right side represents co-transfections. [file 13045_2015_166_MOESM4_ESM.tif]

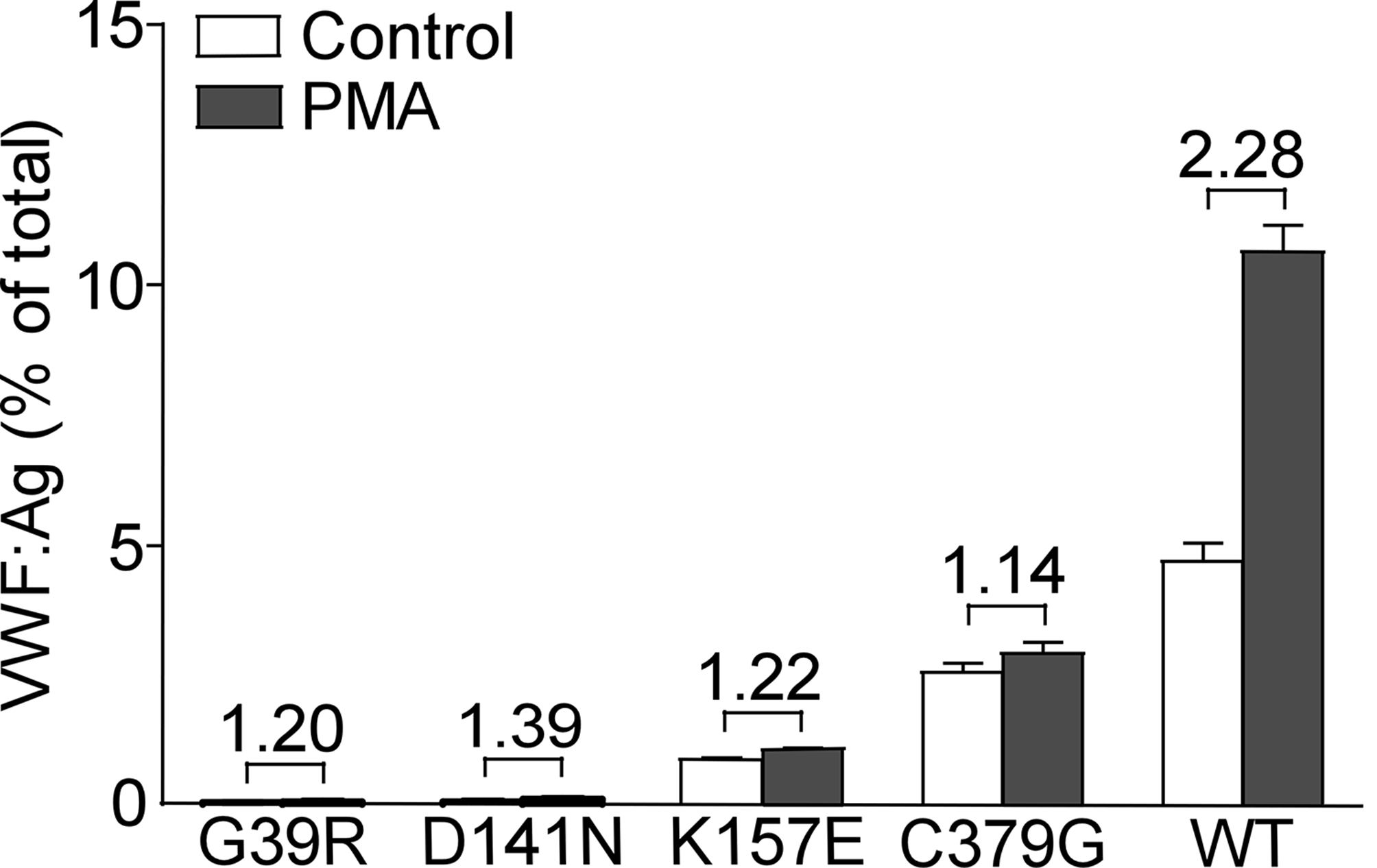

Supplement: Additional file 5: Figure S4. — Decreased regulated secretion of mutant rVWF. Forty-eight hours post-transfection, HEK293 cells were rinsed and changed to release media with or without PMA (control). After further 60 min incubation, VWF levels were determined by ELISA in the release media and cell lysates. Each bar represents VWF secretion in the medium as a percentage of total VWF (medium plus lysate). Error bars indicate standard deviation of triplicate samples. The numbers above the bars indicate the fold increase of stimulated release compared with the control samples. [file 13045_2015_166_MOESM5_ESM.tif]
